# Host Pathogen Relations: Exploring Animal Models for Fungal Pathogens

**DOI:** 10.3390/pathogens3030549

**Published:** 2014-06-30

**Authors:** Catherine G. Harwood, Reeta P. Rao

**Affiliations:** Biology and Biotechnology Department, Worcester Polytechnic University, Worcester, MA 01605, USA; E-Mail: cgharwood@wpi.edu

**Keywords:** fungal pathogens, *Candida*, *Cryptococcus*, *Histoplasma*, *Aspergillus*, model host systems

## Abstract

Pathogenic fungi cause superficial infections but pose a significant public health risk when infections spread to deeper tissues, such as the lung. Within the last three decades, fungi have been identified as the leading cause of nosocomial infections making them the focus of research. This review outlines the model systems such as the mouse, zebrafish larvae, flies, and nematodes, as well as *ex vivo* and *in vitro* systems available to study common fungal pathogens.

## 1. Introduction

**Figure 1:**
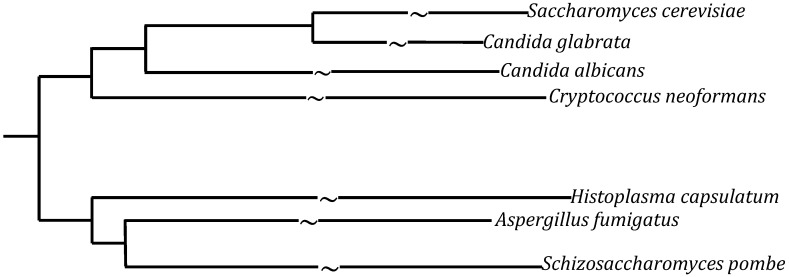
Relative relatedness of the genera discussed in this review [3]. *S. cerevisiae* and *S. pombe* are used as references for comparison. The arm lengths on the phylogenetic tree are representative and not actual evolutionary distances.

Fungi are ubiquitous and can grow on the skin, mucous membranes, and intestinal tracts. They are also found in the soil and on plants, trees, and other vegetation. Although not all fungi are pathogenic, some can cause serious disease and pose a significant public health risk. Within the last three decades, fungi have been identified as the leading cause of nosocomial infections [1,2] especially among immunocompromised patients. This review outlines the host systems available to study some of the common fungal pathogens, of the genera *Candida*, *Cryptococcus*, *Histoplasma*, and *Aspergillus* (Figure 1).

### 1.1. Candida

Yeasts that belong to the genus *Candida* can cause both superficial and invasive infections. Superficial infections include oropharyngeal infection or “thrush”, vaginitis (commonly called “yeast infection”), and diaper rash. While superficial infections are often persistent, they are usually not life threatening. However, when *Candida* invades the blood stream, the infection becomes systemic and is often deadly [4,5]. Fungi in the *Candida* genus have emerged as major human pathogens, becoming the fourth leading cause of bloodstream infection (BSI) in hospitals around the United States, with 7000–28,000 annual reports of BSI with a *Candida* [2]. Some strains, specifically *C. albicans,* are becoming increasingly resistant to conventional antifungal treatments [2]. For example, in 2013 the United States Centers for Disease Control (CDC) reported that fluconazole resistant *Candida albicans* is a serious threat and is responsible for approximately 3,400 cases, both superficial and blood stream, annually [4]. The life style and virulence strategies used by *C. albicans* are the subject of intense research [2]. A BSI often begins with a biofilm on medical devices, such as dental implants, catheters, heart valves, bypass grafts, ocular lenses, artificial joints, and central nervous system (CNS) shunts [1]. In persons whose gastrointestinal tract is heavily colonized by *C. albicans,* it is thought to invade the epithelium in the villi and cross into the blood stream. The most common non-*albicans* infections seen are caused by the emerging opportunistic pathogens *C. parapsilosis, C. tropicalis,* and *C. glabrata.*

*C. parapsilosis* is also capable of forming biofilms on catheters, which can lead to systemic infection if the catheter is placed before removing the yeast [1]. *Candida glabrata,* evolutionarily a distant relative of *C. albicans*, was once considered nonpathogenic [1]. With the increasing incidence of AIDS and other immunosuppressive agents, *C. glabrata* infections have also increased. *C. glabrata* is the second or third most common causative agent of superficial and systemic *Candida* infections. It is often difficult to treat because it is resistant to many azole antifungal agents [6]. *Candida tropicalis* is the fourth most common species of the *Candida* genus [2]. It is common among patients with hematologic malignancies. It is sensitive to azole drugs and other antifungal agents, which have decreased the incidence of *C. tropicalis* within the US. However, worldwide, its incidence continues to increase, which makes it a candidate for study [2].

### 1.2. Cryptococcus

There are approximately thirty different species of *Cryptococcus*, the most prevalent pathogen being *C. neoformans*. *C. neoformans* can be found in soil throughout the world. Humans become infected by inhaling microscopic spores, which can cause anywhere from mild symptoms to serious lung infections [7]. *C. neoformans* is also responsible for cryptococcal meningoencephalitis, which occurs in immunocompromised individuals. It can be found worldwide and is commonly fatal in Sub-Saharan Africa [8]. *C. gattii*, a less known species of *Cryptococcus*, can infect immunocompetent hosts and is found most prominently in tropical and subtropical areas. However, in 1999, there was an outbreak of *C. gattii* on Vancouver Island, Canada. This geographic expansion in its range is interesting and the focus of intense epidemiological studies [9].

### 1.3. Histoplasma

Histoplasmosis, caused by the fungus *Histoplasma capsulatum* is a rare infection but is important to study because of its wide host range, typically mammals. *H. capsulatum* is thought to enter a latent stage that can be reactivated to active histoplasmosis when the patient has a weakened immune system [10] even several years post exposure. *H. capsulatum* is found in the environments most commonly associated with bird and bat droppings where nitrate content is high. Lung infections can occur on inhalation of airborne spores, which produces pneumonia-like symptoms [11].

### 1.4. Aspergillus

The genus *Aspergillus* is a fungi common in the environment, where most people are exposed to the aerosolized spores. Healthy individuals are able to expel the spores from their airways, but immunocompromised individuals are unable to, thus causing serious infection. Initial infection occurs in the lungs, but can spread to almost any organ. The two species that are commonly found associated with disease are *A. fumigatus* and *A. flavus* [12]. During infection, *A. fumigatus* causes significant inflammation and necrosis of the lung tissue through hyphal growth, which restricts oxygen availability in the tissue [13]. *A. flavus* is the second leading cause of invasive aspergillosis and the most common for superficial infections. In certain hospitals though, it is more common in the air than *A. fumigatus*, for reasons that are unclear [10].

## 2. Animal Models

**Figure 2:**
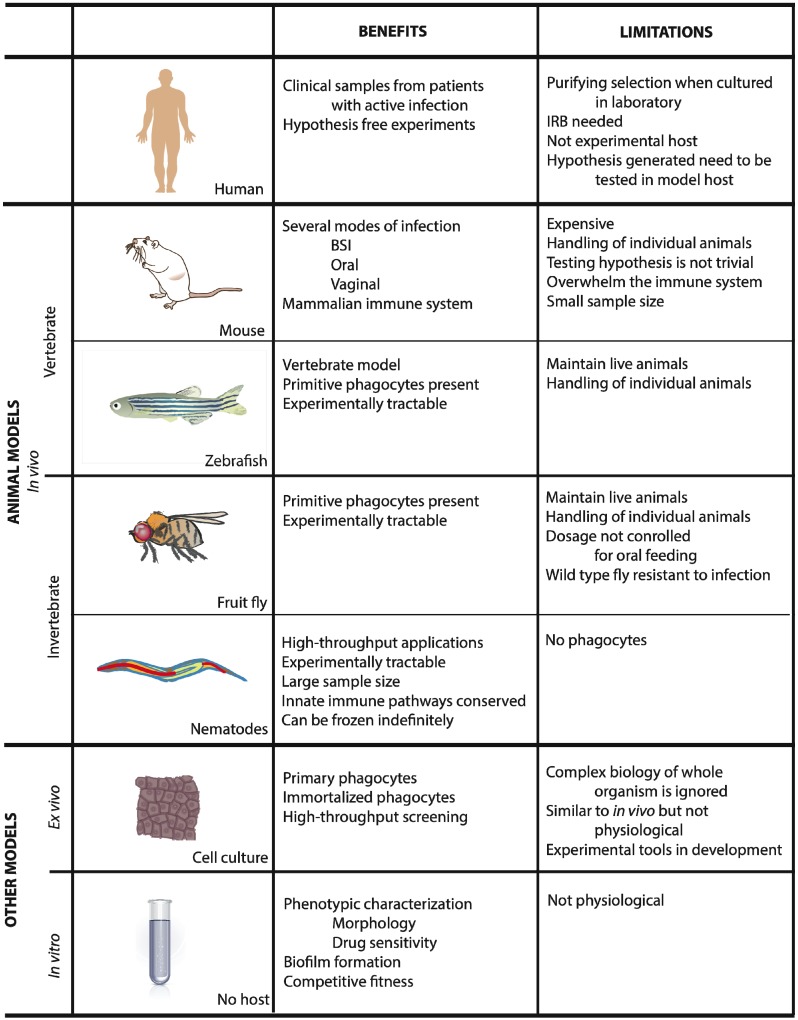
Summary of the various model hosts used to study fungal pathogens.

Several animal hosts have been used to study fungi that cause these common infections (Figure 2). Each model has its limitations but can yield useful information. Therefore, it is crucial that the researcher chooses an appropriate model best suited to address the experimental hypothesis being tested. The most common host systems are mice, zebrafish larvae, fruit flies, and nematodes.

### 2.1. Mus Musculus

The murine model for infection is well developed because of its direct implication for human systems. The mouse has been used to model oral, vaginal, and systemic infections, which have been shown to mimic human infection [14,15,16].

Approximately 90% of HIV and AIDS patients experience an episode of oropharyngeal candidiasis or “thrush.” Rabbits and rats have also been used to model oral infections but these animal models did not accurately mimic local symptoms of oral infection in humans [16]. A mouse model showed typical lesions, associated with disease, allowing for more accurate quantification of the disease burden [14]. This mouse model for oral *Candida* infection has significantly contributed to our understanding of the disease ([17,18,19]). For example, it has been used to show that Th17 helper T cells and their secreted cytokine IL-17 have a protective role against oral candidiasis. Oral candidiasis can lead directly to deep tissue (esophageal or stomach) or systemic *Candida* infections [20].

Recurring vulvovaginitis, which affects approximately 5%–10% of healthy women of childbearing age, is primarily caused by *C. albicans* or *C. glabrata*. A mouse model to understand the effects of vaginitis has been developed [21]. Women with diabetes are particularly at risk for developing vaginitis. This is an important correlation between vulvovaginitis and *Candida* strains, which was used to validate this model. A diabetic mouse model was more susceptible to *C. glabrata* vaginal infections. Greater than 85% of mice showed organisms in lavage fluid and a 25% mortality rate by day 10 [6].

Introducing fungi directly into the tail vein of mice, mimicking a BSI, is widely used [22,23] and is considered “the Gold Standard” in the field. This model is used to verify genetic determinants of virulence that have been identified using alternate methods. It is also useful for testing the efficacy of antifungal therapies. Because of resistance, new antifungals are in high demand. In order to model antifungal resistance in mammals, venous tail injection of *Candida* strains including *albicans* and *tropicalis* as well as *Aspergillus fumigatus* have been used. The mouse model allows for multiple controlled doses of the antifungals [22].

While the mouse is the smallest mammal that mimics many fungal diseases of humans, the model has serious limitations. These include: ethical considerations that prohibit testing on large numbers of animals; the small sample size (n) makes it challenging to observe small differences; the fungal burden introduced in the blood steam via the tail vein can overwhelm the immune system of the mouse, while lower fungal burdens escape detection or do not yield statistically reproducible results; the mouse is also not experimentally tractable for hypothesis-driven research; lastly, knockouts are expensive, time consuming and laborious. Therefore, to test mechanistic hypotheses involving multiple genes, other experimentally tractable facile models such as zebrafish, fruit flies, and nematodes, are used.

### 2.2. Zebrafish

Zebrafish have been used extensively to study developmental biology [24]. Recently, they have been used as vertebrate model hosts to study the interface between a fungal pathogen and the host immune system, Zebrafish express cytokines, macrophages, neutrophils, dendritic cells, mast cells, eosinophil, T-cells, and B-cells that are evolutionarily related to those of humans [25]. Phagocytes are the primary line of defense against fungal infections. In defense, *C. albicans* has been shown to hyphae that can escape the phagosomes [26,27,28].

Phagocytes, neutrophils and macrophages, ingest the fungi and inactivate, via multiple mechanisms, including reactive oxygen species [29,30] and reactive nitrogen species [31]. Patients with neutropenia or defects in NADPH oxidase present an increase in infections with *C. albicans* [23,32].

Zebrafish larvae are transparent, and therefore, dissemination of the infection can be microscopically tracked [33] offering a unique perspective compared to adult fish. Furthermore, larval innate immunity can be efficiently modulated using antisense morpholino mediated gene knockdown [34].

The dimorphic transition between hyphal and yeast from cells is an important virulence determinant for *C. albicans*. The *tup1* mutant of *C. albicans* is locked in a filamentous form and is avirulent in a mouse model [35]. As a corollary, the *cph1efg1* null mutant of *C. albicans* is restricted to yeast form cells under most conditions [36,37], due to mutations in the master regulator of hyphal growth. This mutant has been shown to be less virulent in mice, fruit flies, and nematodes [38,39,40]. Zebrafish were exposed to the *cph1efg1* mutant as a test of virulence. Using fluorescent imaging in the transparent larvae, the non-hyphal forming mutant strain was shown to be less virulent in zebrafish larva [38]. Imaging the macrophages and neutrophils infected with *C. albicans* gave an *in vivo* view of phagocytosed *C. albicans* surviving and/or dividing. This *in vivo* evidence leads to the conclusion that production of reactive oxygen species by the phagocyte’s NADPH oxidase, which has previously been implicated in mice and nematodes [41,42], also provides resistance to *C. albicans* in zebrafish [33].

While zebrafish are a smaller vertebrate alternative to the mouse model, it has certain limitations. Like the mouse, propagation of the fish requires a substantial capital investment for a facility and the personnel to maintain them. Furthermore, zebrafish are typically infected via microscopic injection which requires handling of individual animals, and is thus labor intensive.

### 2.3. Drosophila Melanogaster

*Drosophila melanogaster* is a well-developed experimental system used to model many aspects of biology [43]. It has a short life cycle, easy to maintain and manipulate with a large collection of mutant lines. *D*. *melanogaster* has been used as a host for gram-positive and negative bacteria [44] and more recently, fungi [45]. *D*. *melanogaster* immunity has been shown to possess extensive conservation with mammalian innate immunity pathways, which make it a strong model system. *Drosophila* hemocytes mimic phagocytes in vertebrates, but they lack an acquired immune system. The *D*. *melanogaster* innate immune response is characterized by the production and release of stable antimicrobial peptides by the fat body.

Direct injection of the fungal pathogen into the haemolymph results in a systemic infection and allows for precise calculation of fungal burden. Wild type flies are able to contain a systemic infection but unable to kill injected yeast. Flies with a mutation in the Toll pathway succumb to systemic fungal infections. This immunodeficient fly model has been used as a host for the study of infectious agents [44,46,47,48].

The infectious agent can also be introduced into the normal *Drosophila* diet, although there is no way to accurately monitor intake [39]. The feeding protocol is amenable to scale up studies for unbiased mutant screening to uncover novel virulence mechanisms. In addition, antifungal agents can be mixed in with their normal diet for studies of drug efficacy.

### 2.4. Caenorhabditis Elegans

Like *D. melanogaster*, *Caenorhabditis elegans* have been used extensively as a model system to study various biological phenomena, due to many positive aspects including a rapid lifecycle, its completely sequenced genome, and genetically identical progeny (due to self-fertilization) and a transparent cuticle that facilitates whole animal microscopy [49]. More recently, it has been used an animal model for the study of infectious disease [29,50,51,52]. Nematodes are exposed to the infectious agent in their diet, therefore multiplicity of infection is not easily determined [53]. Fungi concentrate distal to the pharyngeal grinder, and infected worms show a distended gastrointestinal system [29,30,53].

*C. elegans* has been a host model for many gram-positive and gram-negative bacterial infections such as *Salmonella typhimurium, Pseudomonas aeruginosa,* and *Staphylococcus aureus*. Certain long-lived *C*. *elegans* mutants show immunity to these bacterial infections [54]. Recently, *C. elegans* was used as a host to study fungal diseases but more importantly discoveries made in worms, recapitulate virulence patterns in mouse and human infection [53]. For example, several virulence genes, including protein kinase A (PKA), protein kinase R (PKR), and G protein alpha (GPA) were first identified in *C. neoformans*, which modulates virulence in *C*. *elegans* [55]. These same genes were later shown to be important for *Cryptococcus* infection in mammals [53]. Disrupting PKA increases virulence of *C. neoformans* in mice [56], a phenomenon, which was replicated in *C. elegans*. 

Infected nematodes show a deformity in the post anal region (Dar), which is visible four to five days post infection [29,52]. Other disease markers such as, swelling in the vulvar region and intestinal distention, have been reported for both fungal and bacterial infections [29,52]. Ingestion of yeast can be microscopically monitored over time using fluorescently marked *C. albicans* [29], confirming that accumulation of fungi is the likely cause of intestinal distension.

These disease markers can be used to study the progression of disease, while survival assays provides a quantitative measure of disease and death. As a simple starting model, *C. elegans* is a quick and inexpensive way to study virulence of many fungal species. However, like *D. melanogaster*, the results often need to be verified in a mammalian system, like mice.

The nematode is a convenient model especially to study innate immunity since facets of its innate immune system are conserved in humans [29,30] and host genes can be easily modulated via RNAi [57]. Furthermore, infections do not require handling of animals and, therefore, can be performed in a high throughput fashion. Another advantage is that worms can be frozen (indefinitely) and, therefore, do not require maintenance of live cultures.

However, nematodes cannot be propagated at the human body temperature of 37 °C. Therefore, the larvae of the greater wax moth *Galleria mellonella* are used to study virulence at 37 °C. Specifically, the virulence of certain clinical isolates of *C. albicans* has been correlated between the *G. mellonella* infections and murine models, lending credibility to the system. In addition, several bacterial strains, such as *P. aeruginosa,* have been tested in the moth system. In contrast to other readily available, inexpensive systems, like *D. melanogaster* and *C. elegans*, *G. mellonella* has to be tediously injected one at a time and its genome has not been sequenced, which makes knockouts and other genetic manipulations more difficult [38].

## 3. *Ex Vivo* Systems

*Ex vivo* systems for studying fungal infections include phagocytes in culture and tissue explants. Phagocytes are our primary defense against fungal pathogens. Macrophages and neutrophils play a central role in antifungal immunity by ingesting and killing pathogens [26,58,59,60]. They are also responsible for initiating adaptive immune responses.

Cultured macrophages can include both primary and immortalized cells. Immortalized cell lines such as the RAW264.7 and J774 are robust and can withstand harsh treatments such as trypsinization and scraping while still proliferating. Immortalized macrophages have been instrumental in uncovering aspects of *Candida* virulence such as hyphae formation, extracellular lipase, and proteinase production [31]. However, the multiple passages and harsh growth and handling conditions of immortalized macrophages specifically select them to be more robust at the expense of other properties that might be important for immunity. Therefore, primary macrophages are considered better than immortalized macrophages even though they are more difficult to extract and maintain [31].

Both immortalized murine macrophage and primary human macrophage hosts were infected with *C. parapsilosis.* The fungi exhibited varying degrees of virulence when exposed to each type of macrophage [31]. *C. parapsilosis* exhibited the highest resistance to killing by primary human macrophages, compared to *C. orthopsilosis* and *C. metapsilosis*. However, this difference was not reflected in the murine virulence model. In spite of these differences, cultured cell lines are useful and can complement live, whole animal studies.

Transcriptional profiling of *C. albicans* within phagosomes has been performed [20]. Strains have been shown to quickly adapt and alter their transcriptional profile in many circumstances related to infection, such as response to stress, iron deprivation, and interaction with macrophages. In order to understand *Candida* infection, the authors used *in vivo* and *ex vivo* techniques to understand the genes associated with infection in parenchymal organs (e.g., liver). For example, an *ex vivo* tissue explant of porcine liver infected tissue was shown to mimic murine model of *in vivo* infection [20]. *C. albicans* was directly injected into both mice and pig livers. *Ex vivo* tissue explants from both exhibited a similar extent of deep tissue invasion [20]. Excised murine vaginal mucosal tissue is also used as a substrate to study vaginal infections [61]. These *ex vivo* systems provide a rapid and simple method for optimizing conditions prior to *in vivo* assays [62].

## 4. *In Vitro* Systems

Viewing a fungal phenotype on an agar plate is a “host-free” method to study fungal virulence. For example, *C*. *albicans* mutants such as *efg1*−/− and *cph1*−/− showed reduced hyphal growth on agar media, and the double knockout *efg1*−/− *cph1*−/− does not form hyphae under most *in vitro* conditions. This double knockout mutant was later shown to be avirulent in a murine model, which linked hyphal growth and virulence [26]. Since then, many mutants with defects in dimorphic transition have been shown to be attenuated in virulence. This *efg1*−/− *cph1*−/− double mutant has been used extensively to validate other host models [29,33].

Chemical sensitivity *in vitro* can be used as a proxy for pathways that are important *in vivo*. Fungal response to important aspects of the human innate immune system such as reactive oxygen species can be mimicked *in vitro* using menadione and hydrogen peroxide [63]. Cell wall stressors such as SDS, caffeine, and calcofluor [63] have been used *in vitro* to study the effects on the cell wall. Since human cells do not possess cell walls, they are good targets for drugs, like capsofungin [63]. Other drug sensitivity assays can be modeled *in vitro* by plating fungi on drug-infused plates [63]. The role of metals can be mimicked *in vitro* by using scavengers or chelators *in vitro*. For example, the *C. albicans* transcription factor Hap43 is activated by iron deficiency *in vivo* [64] and is also required for growth upon iron-deprivation *in vitro* [63,64,65]. Hap43 also regulates virulence, and mice infected with *hap43*−/− strains showed attenuated virulence [65]. The pathways regulating iron homeostasis are complicated, but *in vitro* techniques may be used to fine tune regulatory networks.

Biofilm formation is a virulence determinant that is well suited to study in a host free environment. Biofilms are typically made up of multiple microbial communities, which stick to each other and are an abiotic surface [66,67]. The microbes secrete extracellular materials [68] and are important in the context of infection because they are drug resistant. For example, *C. albicans* is thought to gain access to the blood stream via plastic devices such as a central line, catheter or other implanted devices. The mortality rate for patients with catheter-related BSI is around 41 percent [68].

Formation of biofilm proceeds three phases: the early phase where the fungal communities attach to the surface, grow and aggregate; the intermediate phase where the extracellular matrix, a predominantly noncellular material, covers fungal microcolonies and is secreted into the milieu [68]. *C. albicans* also transition into the hyphal form; finally, during the maturation phase, the extracellular material grows and planktonic yeast cells bud off and disseminate.

*In vitro* models can also be used to study reconstituted epithelial cells in order to model human infection. The role of certain virulence genes in fungal infection can first be modeled *in vitro* on reconstituted epithelial cells before using more expensive *in vivo* models. For example, the secreted aspartyl proteinases (SAP) of *C. albicans* have been linked to virulence in mucosal infection in clinical isolates of patients with chronic vaginitis [69]. This family of 10 enzymes was first studied using reconstituted vaginal epithelial cells in order to link them to epithelial damage that occurs during disease. The 10 *SAP* genes are differentially expressed during infection. Specifically, *SAP1*, *SAP6*, *SAP9*, and *SAP10* showed a stronger signal in conjunction with mucosal lesions [69]. These *SAP* genes showed strong signals in both *in vitro* models as well as *in vivo* samples from patients with vaginal infections. These lesions showed severe damage in the epithelial cells including: edema, vacuolization, and detachment of keratinocytes. Differential SAP expression has also been linked to “thrush” infections [69]. Despite this, they are not identical to those present in vaginal infections [69].

*In vitro* systems extensively used to study fungal virulence to get mechanistic clues but often have to be validated in an animal model to account for the complex biology in the host environment.

## 5. Conclusions

Several models, including *in vivo*, *ex vivo*, or *in vitro* systems, have been used to study fungal disease. All models have limitations but are useful for generating testable hypotheses. The key is to choose a simple model that can fully address the scientific question being addressed, yet not so simple that the complex and interesting biology is ignored. For example, biofilms and certain drug sensitivities’ assays can be first performed *in vitro*, but then must be verified *in vivo*. Simple and inexpensive systems such as *C. elegans* and *D. melanogaster* can unveil the building blocks for infection, which can then be followed up in mammalian systems. Opportunistic fungal infections are increasing in the United States and around the world. It is important that the host system chosen have implications for furthering the knowledge on human-pathogen interactions.

## References

[1] Centers for Disease Control and Prevention Fungal Disease.

[2] Pfaller M.A., Diekema D.J. (2007). Epidemiology of invasive candidiasis: A persistent public health problem. Clin. Microbiol. Rev..

[3] Birren B.G.F.E.L. (2003). Fungal Genome Initiative: A White Paper for Fungal Comparative Genomics.

[4] Centres for Disease Control and Prevention (2013). Fluconazole-Resistant Candida. Antibiotic Resistance Threats in the United States.

[5] Centers for Disease Control and Prevention Candidiasis. http://www.cdc.gov/fungal/diseases/candidiasis/.

[6] Fidel P.L., Vazquez J.A., Sobel J.D. (1999). Candida glabrata: Review of epidemiology, pathogenesis, and clinical disease with comparison to *C. albicans*. Clin. Microbiol. Rev..

[7] Centers for Disease Control and Prevention *C. neoformans* cryptococcosis. http://www.cdc.gov/fungal/diseases/cryptococcosis-neoformans/.

[8] Price M.S., Betancourt-Quiroz M., Price J.L., Toffaletti D.L., Vora H., Hu G., Kronstad J.W., Perfect J.R. (2011). Cryptococcus neoformans requires a functional glycolytic pathway for disease but not persistence in the host. MBio.

[9] Fraser J.A., Giles S.S., Wenink E.C., Geunes-Boyer S.G., Wright J.R., Diezmann S., Allen A., Stajich J.E., Dietrich F.S., Perfect J.R. (2005). Same-sex mating and the origin of the Vancouver Island Cryptococcus gattii outbreak. Nature.

[10] Rappleye C.A., Engle J.T., Goldman W.E. (2004). RNA interference in Histoplasma capsulatum demonstrates a role for α-(1,3)-glucan in virulence. Mol. Microbiol..

[11] Centers for Disease Control and Prevention Histoplasmosis. http://www.cdc.gov/fungal/diseases/histoplasmosis/.

[12] Willger S.D., Puttikamonkul S., Kim K.-H., Burritt J.B., Grahl N., Metzler L.J., Barbuch R., Bard M., Lawrence C.B., Cramer R.A. (2008). A sterol-regulatory element binding protein is required for cell polarity, hypoxia adaptation, azole drug resistance, and virulence in Aspergillus fumigatus. PLoS Pathog..

[13] Hedayati M.T., Pasqualotto A.C., Warn P.A., Bowyer P., Denning D.W. (2007). Aspergillus flavus: Human pathogen, allergen and mycotoxin producer. Microbiology.

[14] Conti H.R., Shen F., Nayyar N., Stocum E., Sun J.N., Lindemann M.J., Ho A.W., Hai1 J.H., Yu J.J., Jung J.W. (2009). Th17 cells and IL-17 receptor signaling are essential for mucosal host defense against oral candidiasis. J. Exp. Med..

[15] Fidel P.L., Cutright J.L., Tait L., Sobel J.D. (1996). A murine model of Candida glabrata vaginitis. J. Infect. Dis..

[16] Takakura N., Sato Y., Ishibashi H., Oshima H., Uchida K., Yamaguchi H., Abe S. (2003). A novel murine model of oral candidiasis with local symptoms characteristic of oral thrush. Microbiol. Immunol..

[17] Fidel P.L. (2002). Distinct protective host defenses against oral and vaginal candidiasis. Med. Mycol..

[18] Koh A.Y., Köhler J., Coggshall K.T., Rooijen N.V., Pier G.B. (2008). Mucosal damage and neutropenia are required for Candida albicans dissemination. PLoS Pathog..

[19] Kumamoto C.A., Vinces M.D. (2005). Alternative Candida albicans lifestyles: Growth on surfaces. Annu. Rev. Microbiol..

[20] Thewes S., Kretschmar M., Park H., Schaller M., Filler S.G., Hube B. (2007). *In vivo* and *ex vivo* comparative transcriptional profiling of invasive and non-invasive Candida albicans isolates identifies genes associated with tissue invasion. Mol. Microbiol..

[21] Wormley F.L., Steele C., Wozniak K., Fujihashi K., McGhee J.R., Fidel P.L. (2001). Resistance of T-Cell Receptor δ-Chain-Deficient Mice to Experimental *Candidaalbicans* Vaginitis. Infect. Immun..

[22] Ikeda F., Wakai Y., Matsumoto S., Maki K., Watabe E., Tawara S., Goto T., Watanabe Y., Matsumoto F., Kuwahara S. (2000). Efficacy of FK463, a new lipopeptide antifungal agent, in mouse models of disseminated candidiasis and aspergillosis. Antimicrob. Agents Chemother..

[23] Lopez-Berestein G., Hopfer R.L., Mehta R., Mehta K., Hersh E.M., Juliano R.L. (1984). Liposome-encapsulated amphotericin B for treatment of disseminated candidiasis in neutropenic mice. J. Infect. Dis..

[24] Van der Sar A.M., Appelmelk B.J., Vandenbroucke-Grauls C.M.J.E., Bitter W. (2004). A star with stripes: Zebrafish as an infection model. Trends Microbiol..

[25] Tobin D.M., May R.C., Wheeler R.T. (2012). Zebrafish: A see-through host and a fluorescent toolbox to probe host–pathogen interaction. PLoS Pathog..

[26] Lo H.-J., Köhler J.R., DiDomenico B., Loebenberg D., Cacciapuoti A., Fink G.R. (1997). Nonfilamentous C. albicans mutants are avirulent. Cell.

[27] Liu H., Kohler J., Fink G.R. (1994). Suppression of hyphal formation in Candida albicans by mutation of a STE12 homolog. Science.

[28] Lorenz M.C., Bender J.A., Fink G.R. (2004). Transcriptional response of Candida albicans upon internalization by macrophages. Eukaryot. Cell.

[29] Jain C., Pastor K., Gonzalez A.Y., Lorenz M.C., Rao R.P. (2013). The role of Candida albicans AP-1 protein against host derived ROS in *in vivo* models of infection. Virulence.

[30] Jain C., Yun M., Politz S.M., Rao R.P. (2009). A pathogenesis assay using Saccharomyces cerevisiae and Caenorhabditis elegans reveals novel roles for yeast AP-1, Yap1, and host dual oxidase BLI-3 in fungal pathogenesis. Eukaryot. Cell.

[31] Németh T., Tóth A., Szenzenstein J., Horváth P., Nosanchuk J.D., Grózer Z., Tóth R., Papp C., Hamari Z., Vágvölgyi C., Gácser A. (2013). Characterization of Virulence Properties in the *C. parapsilosis Sensu Lato* Species. PLoS One.

[32] Robbins S.L., Kumar V., Cotran R.S. (2010). Robbins and Cotran Pathologic Basis of Disease.

[33] Brothers K.M., Newman Z.R., Wheeler R.T. (2011). Live imaging of disseminated candidiasis in zebrafish reveals role of phagocyte oxidase in limiting filamentous growth. Eukaryot. Cell.

[34] Summerton J., Weller D. (1997). Morpholino antisense oligomers: Design, preparation, and properties. Antisense Nucleic Acid Drug Dev..

[35] Braun B.R., Johnson A.D. (1997). Control of filament formation in Candida albicans by the transcriptional repressor TUP1. Science.

[36] Rao R.P., Hunter A., Kashpur O., Normanly J. (2010). Aberrant synthesis of indole-3-acetic acid in Saccharomyces cerevisiae triggers morphogenic transition, a virulence trait of pathogenic fungi. Genetics.

[37] Kumamoto C.A., Vinces M.D. (2005). Contributions of hyphae and hypha-co-regulated genes to Candida albicans virulence. Cell. Microbiol..

[38] Brennan M., Thomas D.Y., Whiteway M., Kavanagh K. (2002). Correlation between virulence of Candida albicans mutants in mice and Galleria mellonella larvae. FEMS Immunol. Med. Microbiol..

[39] Chamilos G., Lionakis M.S., Lewis R.E., Lopez-Ribot J.L., Saville S.P., Albert N.D., Halder G., Kontoyiannis D.P. (2006). Drosophila melanogaster as a facile model for large-scale studies of virulence mechanisms and antifungal drug efficacy in Candida species. J. Infect. Dis..

[40] Pukkila-Worley R., Ausubel F.M., Mylonakis E. (2011). Candida albicans infection of Caenorhabditis elegans induces antifungal immune defenses. PLoS Pathog..

[41] Frohner I.E., Bourgeois C., Yatsyk K., Majer O., Kuchler K. (2009). Candida albicans cell surface superoxide dismutases degrade host-derived reactive oxygen species to escape innate immune surveillance. Mol. Microbiol..

[42] Chávez V., Mohri-Shiomi A., Maadani A., Vega L.A., Garsin D.A. (2007). Oxidative stress enzymes are required for DAF-16-mediated immunity due to generation of reactive oxygen species by Caenorhabditis elegans. Genetics.

[43] Kornberg T.B., Krasnow M.A. (2000). The Drosophila genome sequence: Implications for biology and medicine. Science.

[44] Leclerc V., Reichhart J.M. (2004). The immune response of Drosophila melanogaster. Immunol. Rev..

[45] Alarco A.-M., Marcil A., Chen J., Suter B., Thomas D., Whiteway M. (2004). Immune-deficient Drosophila melanogaster: A model for the innate immune response to human fungal pathogens. J. Immunol..

[46] Glittenberg M.T., Kounatidis I., Christensen D., Kostov M., Kimber S., Roberts I., Ligoxygakis P. (2011). Pathogen and host factors are needed to provoke a systemic host response to gastrointestinal infection of Drosophila larvae by Candida albicans. Dis. Models Mech..

[47] Lemaitre B., Nicolas E., Michaut L., Reichhart J.M., Hoffmann J.A. (1996). The Dorsoventral Regulatory Gene Cassette spätzle/Toll/cactus Controls the Potent Antifungal Response in Drosophila Adults. Cell.

[48] Quintin J., Asmar J., Matskevich A.A., Lafarge M.C., Ferrandon D. (2013). The Drosophila Toll pathway controls but does not clear Candida glabrata infections. J. Immunol..

[49] Stiernagle T. (2006). Maintenance of C. elegans. WormBook. The C. elegans Research Community.

[50] Ewbank J.J. (2002). Tackling both sides of the host–pathogen equation with *Caenorhabditis elegans*. Microbes Infect..

[51] Garsin D.A., Villanueva J.M., Begun J., Kim D.H., Sifri C.D., Calderwood S.B., Ruvkun G., Ausubel F.M. (2003). Long-lived C. elegans daf-2 mutants are resistant to bacterial pathogens. Science.

[52] Hodgkin J., Kuwabara P.E., Corneliussen B. (2000). A novel bacterial pathogen, Microbacterium nematophilum, induces morphological change in the nematode *C. elegans*. Curr. Biol..

[53] Mylonakis E., Ausubel F.M., Perfect J.R., Heitman J., Calderwood S.B. (2002). Killing of Caenorhabditis elegans by Cryptococcus neoformans as a model of yeast pathogenesis. Proc. Natl. Acad. Sci. USA.

[54] Mahajan-Miklos S., Tan M.-W., Rahme L.G., Ausube F.M. (1999). Molecular Mechanisms of Bacterial Virulence Elucidated Using a Pseudomonas aeruginosa–Caenorhabditis elegans Pathogenesis Model. Cell.

[55] Mylonakis E., Idnurm A., Moreno R., Khoury J.E., Rottman J.B., Ausubel F.M., Heitman J., Calderwood S.B. (2004). Cryptococcus neoformans Kin1 protein kinase homologue, identified through a Caenorhabditis elegans screen, promotes virulence in mammals. Mol. Microbiol..

[56] D'Souza C.A., Alspaugh J.A., Yue C., Harashima T., Cox G.M., Perfect J.R., Heitman J. (2001). Cyclic AMP-dependent protein kinase controls virulence of the fungal pathogen Cryptococcus neoformans. Mol. Cell. Biol..

[57] Kim D.H., Rossi J.J. (2008). RNAi mechanisms and applications. Biotechniques.

[58] Rubin-Bejerano I., Fraser I., Grisafi P., Fink G.R. (2003). Phagocytosis by neutrophils induces an amino acid deprivation response in Saccharomyces cerevisiae and Candida albicans. Proc. Natl. Acad. Sci. USA.

[59] Wheeler R.T., Fink G.R. (2006). A drug-sensitive genetic network masks fungi from the immune system. PLoS Pathog..

[60] Lorenz M.C., Fink G.R. (2001). The glyoxylate cycle is required for fungal virulence. Nature.

[61] Harriott M.M., Lilly E.A., Rodriguez T.E., Fidel P.L., Noverr M.C. (2010). Candida albicans forms biofilms on the vaginal mucosa. Microbiology.

[62] Fazly A., Jainb C., Dehnera A.C., Issib L., Lillyc E.A., Alid A., Caod H., Fidel P.L., Raob R.P., Kaufman P.D. (2013). Chemical screening identifies filastatin, a small molecule inhibitor of Candida albicans adhesion, morphogenesis, and pathogenesis. Proc. Natl. Acad. Sci. USA.

[63] Homann O.R., Dea J., Noble S.M., Johnson A.D. (2009). A phenotypic profile of the Candida albicans regulatory network. PLoS Genet..

[64] Hsu P.-C., Yang C.-Y., Lan C.-Y. (2011). Candida albicans Hap43 is a repressor induced under low-iron conditions and is essential for iron-responsive transcriptional regulation and virulence. Eukaryot. Cell.

[65] Baek Y.-U., Li M., Davis D.A. (2008). Candida albicans ferric reductases are differentially regulated in response to distinct forms of iron limitation by the Rim101 and CBF transcription factors. Eukaryot. Cell.

[66] O'Toole G., Kaplan H.B., Kolter R. (2000). Biofilm formation as microbial development. Annu. Rev. Microbiol..

[67] Hogan D.A., Vik Å., Kolter R. (2004). A Pseudomonas aeruginosa quorum-sensing molecule influences Candida albicans morphology. Mol. Microbiol..

[68] Chandra J., Kuhn D.M., Mukherjee P.K., Hoyer L.L., McCormick T., Ghannoum M.A. (2001). Biofilm formation by the fungal pathogen Candida albicans: Development, architecture, and drug resistance. J. Bacteriol..

[69] Schaller M., Bein M., Korting H.C., Baur S., Hamm G., Monod M., Beinhauer S., Hube B. (2003). The secreted aspartyl proteinases Sap1 and Sap2 cause tissue damage in an *in vitro* model of vaginal candidiasis based on reconstituted human vaginal epithelium. Infect. Immun..

